# Diagnostic Challenges of Low-Grade Central Osteosarcoma of Jaw: a Literature Review

**Published:** 2015-06

**Authors:** Seyd Hosein Tabatabaei, Gholamreza Jahanshahi, Farzad Dehghan Marvasti

**Affiliations:** 1 Dept. of Oral and Maxillofacial Pathology, Yazd Dental School, Shahid Sadughi University of Medical Sciences, Yazd, Iran;; 2 Dept. of Oral and Maxillofacial Pathology, Isfahan Dental School, Isfahan University of Medical Sciences, Isfahan, Iran;; 3 Student of Dentistry, Dept. of Oral and Maxillofacial Pathology, Yazd Dental School, Shahid Sadughi University of Medical Sciences, Yazd, Iran;

**Keywords:** Diagnostic Challenges, Low Grade Central Osteosarcoma, Jaw

## Abstract

Low Grade Central Osteosarcoma (LGCO) is a rare subtype of osteosarcoma that is less aggressive than conventional osteosarcoma. The importance of LGCO lies in the fact that regarding microscopic and radiographic features, it occasionally simulates some benign jaw lesions and would consequently be misdiagnosed in many patients.

The present study was conducted to collect the information and descriptive analyses related to ten cases reported between 1987 and 2010, including a sample reported by the authors emphasizing on diagnostic errors and the prevailing misdiagnosis. The aforementioned reports were gathered in full-texts through Google and PubMed search engines.

**Conclusion:**

The results of this study showed that the pathologists should exactly evaluate the clinical, radiographic, and histopathologic features in order to observe the evidence of invasion.

## Introduction

Osteosarcoma is the most common primary malignant bone tumor with nonhematopoietic origin in children and adolescents[1-6] in which the mesenchymal neoplastic cells are able to produce osteoid or immature bone.[7-8] About 4-13% of skeletal osteosarcomas may arise in the jaws.[9-17] Many investigators believe that gnathic osteosarcomas are less aggressive than the long bone tumors.[3] However, many recent clinicopathologic studies suggest that most of gnathic osteosarcomas are among high-grade lesions.[3, 18-20] Low-grade lesions occur rarely; they are divided into two subgroups of low-grade central osteosarcoma (LGCO) and parosteal osteosarcoma.[1, 21] LGCO generally represents 1-2% of all skeletal osteosarcomas.[13, 22] This rare subtype is less aggressive than the other more frequent types, and has a small risk of metastasis capability with fairly good prognosis.[22]

The significance of LGCO lies in the fact that regarding microscopic and radiologic features, it occasionally simulates some benign jaw lesions and is usually misdiagnosed as a fibrous dysplasia or sometimes other gnathic benign proliferations;[22-37] thus the patient would be deprived of proper and sufficient treatment. Inadequate treatment results in recurrence[15] which in turn increases the degree of malignancy and chance of metastasis.[15, 22]

A limited amount of study has been published on gnathic LGCO. With respect to rarity of this lesion in any large series of investigating skeletal osteosarcomas,[28] data related to this subtype is not separately collected.

Therefore, this study reviewed the literature to collect the information and descriptive analyses related to 10 rare cases reported from 1987 to 2010 including a reported sample by the authors in 2010,[37] with emphasis on epidemiologic, radiographic, and microscopic aspects as well as diagnostic errors. The above-mentioned reports were gathered in full-texts throughGoogle and PubMed search engines.

*Epidemiologic Features*

The investigated gnathic LGCO samples were in a wide age range from 18 to 69 years with the mean age of 35.[24, 31-38]Most of the gnathic osteosarcomas have been diagnosed in the third and fourth decades oflife;[3, 39] four cases were in the second decade and six in the third or higher. The mean age of men at the time of diagnosis of the lesion (45 years) was higher than women (25 years);[24, 32-38] however, the mean age of patients was similar to other related studies.[3, 39] Some reports about conventional osteosarcoma have indicated a slight male predominance,[24, 40-45] although others have reported a higher predominance in females.[9, 46-47] In our study of 10 LGCO samples, 7 were female (70%) and 3 were male (30%), representing a female predominance. 

*Clinical Features*

Some studies have shown an equal frequency of gnathic osteosarcomas in both jaws,[9, 46] while some others have reported prevalenceofosteosarcoma in mandible more than the maxilla.[45]

In the investigated LGCO samples, mandibular involvement was twice as much as the maxilla (3.7= maxilla/mandible)[22, 24, 34-38] as well as conventional osteosarcoma.[9, 15, 43, 46, 49] In our study, mandibular lesions showed more tendency to involve the body (5.7 cases)[24, 32, 34-35, 38] and maxillary lesions were common in the alveolar ridge (3.3 cases).[31-33]

The main symptoms of conventional osteosarcoma are pain and swelling[15, 48-50] and the average time interval from presenting symptoms to diagnosis is 3-5 months.[41] However, gnathic LGCO usually appears in form of long-time swelling with no pain.[35] In 70% of cases of this study, the lesion initiated in the form of a painless swelling[24, 32, 34-38] and the interval between symptom expression and diagnosis was 24 months.[24, 31-38]

*Radiographic Features*

Like other body bones,[11, 51-53] gnathic LGCOsmight be radiographically shown as osteolytic, osteoblastic, or mixed lesions with irregular margins.[53] Some specific radiographic features are ill-defined margins and cortical plate destruction with or without invasion into soft tissues, irregular widening of periodontal ligament space and the sunray appearance; however, these features are not always seen.[28, 54]

Based on the radiographic findings, in two cases out of all investigated samples, there were misdiagnosis of a giant cell granuloma[31] and a benign fibro-osseous lesion.[37]

**Figure 1:**
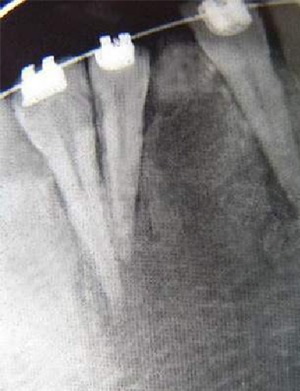
The periapical radiograph of our case showing widening of the periodontal ligament space and a coarse trabecular pattern. These important features were neglected.

In this study, the radiographic features of 7 cases^24,32-34,36-38^ were in favor of malignancy on the onset, but occasionally these important features were neglected (Figure 1). These aggressive radiographic features included destruction of lamina dura and widening of periodontal ligament space (n=3), diffused mixed radiolucency (a cloudy appearance) (n=4), ill-defined margins (n=5), cortical destruction (n=5), and soft tissue invasion (n=3).

*Histopathologic Feature*

LGCO is a well-differentiated malignant neoplasm that is observed as a spindle cell fibroblastic proliferation with low cellularity, without significant atypia; and low mitotic figures (less than 4 mitoses per 10HPF) with a variable osteoid production that may be seen in abundant layers of irregular calcified or scattered layers of osteoid.[28, 36]

With respect to the quality of osteoid distribution, the amount of present collagen, presence of myxoid changes, and the benign lesion it mimics, there are many patterns describing this tumor, including fibrous dysplasia-like, osteoblastoma-like, desmoplastic fibroma-like, parosteal-like, chondromyxoid fibroma-like; however, their occurrence is rare.[26, 28, 54-60]

The LGCO was diagnosed initially based on incisional biopsy in 5 cases [28, 32-33, 35-36] out of all investigated samples. In 3 cases,[24, 31, 34] after excisional biopsy, the initial diagnosis was finally changed from central giant cell granuloma,[31] fibrous dysplasia,[24] and chondromyxoid fibroma[34] to LGCO.

In two investigated samples the lesion that had been first diagnosed as a fibrous dysplasia turned to LGCO with focal area of high-grade osteosarcoma in later recurrences with lesion development in adjacent structures.The proliferation of spindle cells with defined atypia and production of osteoid, chondroid, and formation of irregular osteoid trabeculae were observed.[22, 37] One patient died due to development of the lesion into his vital structures.[24]

*Differential Diagnoses and Misdiagnosis*

The most important feature for correct diagnosis of gnathic LGCO and precluding the misdiagnosis with benign lesions is the observation of clinical symptoms as well as radiographic and histopathologic features of invasion. These features include poor margination of lesion, cortical bone destruction, and invasion into soft tissues.[35] However, histological characteristics, including cellularity amount, cellular atypia, and mitotic activity rate are not very helpful.[36] Therefore, interpretation of small biopsies is very difficult, unless there are definite radiographic evidences showing the presence of an aggressive lesion.[35] Therefore, an excisional biopsy specimen must contain a large and enough part of the tumoral tissue, whilst careful clinical and histopathological evaluation is also required.

Evaluation of cases in this study showed that in most cases (80%) the pathologists were suspicious of a malignant lesion based on radiographic features (Figure 1); although for exact diagnosis, we should not rely on radiographic features alone.[54] Like most reports[27-28,57] among investigated samples, the highest number of misdiagnosis belonged to fibrous dysplasia (2.3 cases).[22, 24, 37]

**Figure 2:**
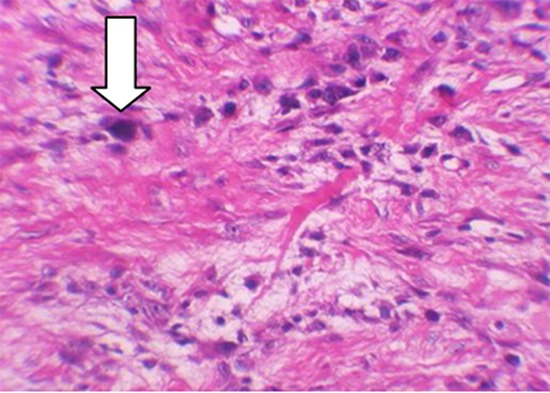
Minimal cellular atypia (white arrow) in our case, constructing the most important diagnosis features since fibrous dysplasia never shows cellular atypia (H&E staining, x40 magnification).

Relatively, low cellular fibroblastic stroma in fibrous dysplasia and LGCO contained spindle cells that produce collagen; however, spindle cells in LGCO have a tendency to arrange in form of crossover groups which are not shown typically in fibrous dysplasia. Although cellular atypia is minimum in LGCO, it holds the most important diagnostic features (Figure 2), since fibrous dysplasia never shows cellular atypia. From the morphological point of view, spindle-shaped nuclei of LGCO are longer and thinner than their fat and small counterparts in fibrous dysplasia. Mitotic figures are hardly ever observed in fibrous dysplasia, whereas in LGCO, at least some mitotic figures are seen.[57]

The most important factor for differentiation of LGCO from fibrous dysplasia is to observe an infiltrative growth pattern. These patterns are described as surrounding pre-existed bone trabeculae with tumor, tumoral cells infiltration into bone marrow, cortex destruction by tumor, and tumor invasion into soft tissues.[37, 57] Based on the quality of ossification in LGCO, three different patterns have been reported so far including fibrous dysplasia-like, parosteal osteosarcoma-like, and desmoid-like.[57]

**Figure 3:**
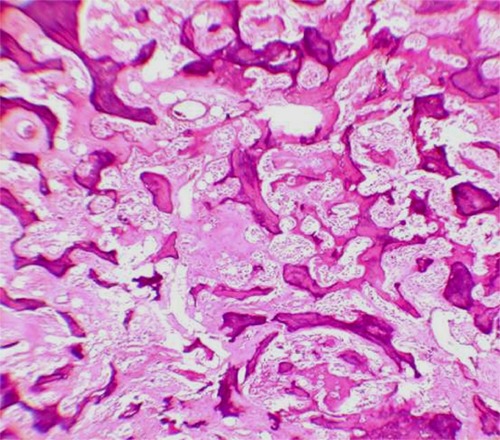
Fibrous dysplasia-like pattern in our case. Coarseness of bone trabeculae was neglected. This feature, in comparison with the branched, delicate, and curvilinear trabeculae in fibrous dysplasia is the most beneficial feature in favor of LGCO (H&E staining, x10 magnification).

Fibrous dysplasia-like type includes irregular spicules of woven bone that sometimes resembles the classic pattern of Chinese script writing.[37, 57] However, compared to the branched, delicate, and curvilinear trabeculae in fibrous dysplasia, the coarseness of bone trabeculae in LGCO is a useful guide[13] (Figure 3). LGCO may resemble parosteal osteosarcoma from the microscopic point of view. Both of these lesions have low cellularity, low amount of mitosis and minimum cellular atypia.[25-26] LGCO may show long and parallel strips of lamellar bone that is microscopically non-diagnosable from parosteal osteosarcoma.[57] In this case, radiographic control is very useful to verify the tumor inter-medullary origin.[26, 35] Desmoid-like pattern has the least prevalence. Desmoplastic fibroma is a benign bony neoplasm formed from fibroblastic and myofibroblastic proliferation in a heavy collagenous stroma and like LGCO, it can destroy cortex and infiltrate into soft tissues.[57] No osteoid production is observed in desmoplastic fibroma and it lacks mitotic figures.[15, 58] Osteoblastoma-like osteosarcoma is a rare variant of osteosarcoma (1.1-1.4%).[26, 59] Histopathologic findings of this tumor are composed of osteoblastoma-like regions of cellular sheets with round nuclei, with or without prominent nucleolus mixed with spindle stroma in conjunction with various amounts of lace-like osteoid. There is no bone formation in this tumor; nevertheless, observing an infiltrative pattern is a diagnostic indication for osteosarcoma .Of course, an infiltrative pattern might be seen rarely in curetted biopsy specimen.[59]

Therefore, while radiographic features suggest an aggressive lesion, the biopsy should contain peripheral areas of the tumor or the overlying cortical bone.[60]

Chondromyxoid fibroma-like osteosarcoma is a completely rare subgroup of low-grade osteosarcoma.[38, 55] Similar to its benign counterpart, chondromyxoid- like osteosarcoma includes some loosely aggregations of stellate, spindle, or polygonal cells in a myxoidstroma. Although the most important diagnostic feature is the production of osteoid by tumor cells, this feature has never been observed in chondromyxoid fibroma.[55, 61]

Solid areas of an aneurismal bone cyst might sometimes be mistaken for LGCO. Aneurismal bone cyst is a benign lesion that hardly ever occurs in craniofacial area. Solid type of this lesion comprises only 5% of all cases and its occurrence in jaws is extremely rare. Aneurismal bone cyst is more cellular than LGCO and has a prominent mitotic activity.[62]

## Conclusion

For early diagnosis of gnathic LGCO and preclusion of misdiagnosis as a benign lesion, the pathologists should exactly evaluate the clinical and radiographic features in order to observe the peripheral border, cortical bone destruction, and invasion to the soft tissues.

An excisional biopsy, which includes overlying soft tissue, cortical bone, and the medullary portion of the lesion is needed and curettage should not be done. By thorough evaluation of various sections of specimen, two important features should be taken into account: 1- Osteoid production by tumor cells 2- Any observable infiltrative pattern. Therefore, the general histomorphologic appearance of the lesion might be more helpful than surveying cellular features.
